# Mutagenesis of the transmembrane domain of the SARS coronavirus spike glycoprotein: refinement of the requirements for SARS coronavirus cell entry

**DOI:** 10.1186/1743-422X-6-230

**Published:** 2009-12-24

**Authors:** Jeroen Corver, Rene Broer, Puck van Kasteren, Willy Spaan

**Affiliations:** 1 Department of Medical Microbiology, Center of Infectious Diseases, Leiden University Medical Center, 2300 RC Leiden, the Netherlands

## Abstract

**Background:**

The spike protein (S) of SARS Coronavirus (SARS-CoV) mediates entry of the virus into target cells, including receptor binding and membrane fusion. Close to or in the viral membrane, the S protein contains three distinct motifs: a juxtamembrane aromatic part, a central highly hydrophobic stretch and a cysteine rich motif. Here, we investigate the role of aromatic and hydrophobic parts of S in the entry of SARS CoV and in cell-cell fusion. This was investigated using the previously described SARS pseudotyped particles system (SARSpp) and by fluorescence-based cell-cell fusion assays.

**Results:**

Mutagenesis showed that the aromatic domain was crucial for SARSpp entry into cells, with a likely role in pore enlargement.

Introduction of lysine residues in the hydrophobic stretch of S also resulted in a block of entry, suggesting the borders of the actual transmembrane domain. Surprisingly, replacement of a glycine residue, situated close to the aromatic domain, with a lysine residue was tolerated, whereas the introduction of a lysine adjacent to the glycine, was not. In a model, we propose that during fusion, the lateral flexibility of the transmembrane domain plays a critical role, as do the tryptophans and the cysteines.

**Conclusions:**

The aromatic domain plays a crucial role in the entry of SARS CoV into target cells. The positioning of the aromatic domain and the hydrophobic domain relative to each other is another essential characteristic of this membrane fusion process.

## Background

The mechanism by which the viral spike proteins mediate the initial stages of membrane fusion is fairly well understood for a number of viruses. Currently, there are three classes of viral fusion proteins recognized. Although structurally unrelated, the viral fusion proteins of all classes refold to establish a conformation that brings the fusion peptide and the transmembrane domain (TMD) in close proximity, thus initializing membrane fusion [1].

As the initial stages of viral membrane fusion, including the refolding of the spike proteins, are well-understood, the exact mechanism by which the membranes merge remains unclear. It is very likely that the transmembrane domains (TMDs) or amino acid residues adjacent to the TMDs of viral fusion proteins, play a role in this process [2]. For instance, Influenza HA molecules that are anchored to a membrane through a GPI anchor in stead of their wild type TMD, are unable to complete the fusion process. Rather, membrane fusion is halted at the hemifusion stage [3-5]. In addition, it has been shown that glycine residues of the TMD of the vesicular stomatitis virus glycoprotein (VSV-G), play a critical role in membrane fusion [6]. Furthermore, it has been shown that the membrane-proximal domain of GP41, the fusion protein of human immunodeficiency virus, is important for fusion activity [7,8]. In particular, aromatic residues have been shown to be involved in the process of fusion pore dilation [7]. Likewise, palmitoylated cysteines, situated in or close to the viral membrane, have been implicated in the fusion process of coronavirus [9-12] and influenza virus [13]. We have shown that the TMDs of coronavirus spike proteins are also crucial for membrane fusion activity. By swapping the TMD of severe acute respiratory syndrome coronavirus (SARS CoV) spike for that of VSV-G, we have shown that both entry of SARS pseudoparticles (SARSpp) and SARS CoV spike protein mediated cell-cell fusion depends on the presence of the TMD of the spike [14].

**Figure 1:**
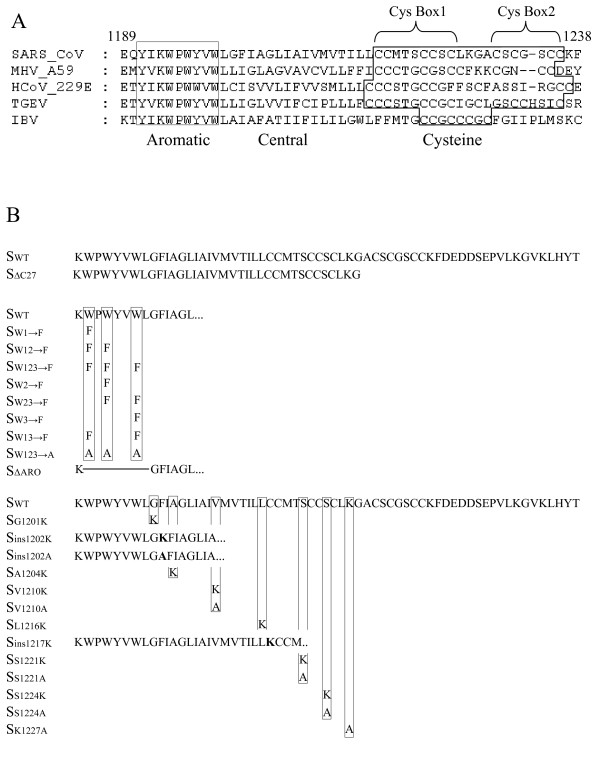
**Characteristics of the SARS coronavirus S protein TMD and adjacent sequences**. A) Alignment of the TMDs and adjacent sequences of coronavirus S proteins. A representative of each coronavirus group is included in the alignment. Numbering is based on the SARS CoV sequence. Abbreviations: MHV, mouse hepatitis virus; HCoV, human coronavirus; TGEV, transmissible gastroenteritis virus; IBV, infectious bronchitis virus. B) Overview of the SARS CoV S mutant proteins that were generated for this study and their nomenclature.

The TMD of the SARS CoV spike protein consists of three domains: 1) a highly conserved N-terminal aromatic (tryptophan) rich stretch, 2) a hydrophobic core sequence and 3) a C-terminal cysteine rich domain. These domains are highly conserved in all coronaviruses (see Figure 1A). In this paper we describe an extensive mutagenesis study of the aromatic domain of SARS CoV S and the effect of these mutations on entry and fusion. In addition, we have tried to map the amino acids that are actually in the membrane by introducing a charged lysine residue at different positions in the predicted TMD region. We measured the capacity of the mutated spike proteins to mediate cell entry of virus like particles using our previously described SARSpp assay [14]. In addition, we determined the oligomeric state of the non-active mutants. The mutants concerning the aromatic domain were further analyzed using fluorescent dye transfer assays. The data presented here show that specific amino acids in or close to the TMD are crucial for membrane fusion activity of SARS CoV.

## Results

Sequence analysis of the TMD of coronavirus spike proteins reveals a high conservation rate. In Figure 1A, an alignment is shown of the transmembrane domains of several coronavirus spike proteins, of which at least one virus of each group is included. Evidently, the aromatic and cysteine domains are conserved between CoVs. To investigate the roles of the aromatic domain in SARS CoV spike-mediated entry, we performed an extensive mutagenesis on the spike TMD. The mutants that were generated are listed in Figure 1B.

### Mutagenesis of the aromatic domain of SARS S

**Figure 2:**
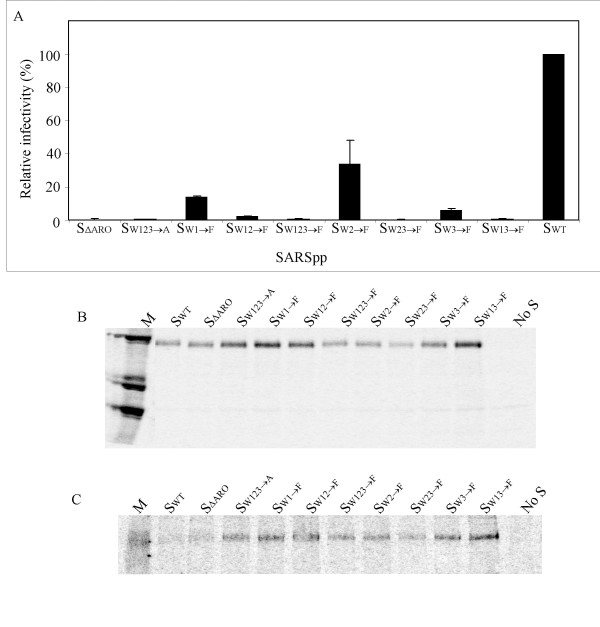
**Capacity of S proteins with mutations in the aromatic domain to mediate entry of SARSpp into VeroE6R cells**. A) Titers of the mutant S containing pseudoparticles are shown as a percentage of wild type S containing pseudoparticles. B) Incorporation of S proteins in pseudoparticles. Equal amounts of [^35^S]-labeled particles, based on RT activity, were subjected to immunoprecipitation, using an S-specific antibody. The precipitates were analyzed using SDS-PAGE and subsequent autoradiography. C) Trimers of S protein were shown by running the precipitates of B on a non reducing gel, without boiling of the samples.

Previously, the aromatic domain of HIV gp41 has been shown to be important for the entry of HIV into target cells [8], in particular for the dilation of fusion pores [7]. Recently, Howard et al. have shown that also the aromatic residues in the juxtamembrane domain of SARS CoV S are important for entry of SARS CoV [15]. By creating mutant SΔARO, we wanted to investigate the importance of the aromatic domain in SARS S-mediated entry. Figure 2 shows that SARSpp, containing S_ΔARO_, are no longer capable of transducing Vero E6 cells, indicating that the aromatic domain is essential for the entry of the SARSpp. Moreover, when all the tryptophan residues in the aromatic domain were replaced for alanines, SARSpp were not infectious either, indicating that the alanines cannot take over the role of the tryptophans during SARS S mediated entry. This suggests a crucial role for the tryptophan residues during SARS S- mediated entry.

Wimley and White have described an index on which the membrane interfaciality of amino acids has been ranked [16]. This index is indicative for the tendency of amino acids to participate in the interface of membranes and water. Tryptophan scores highest on this WW-index. Second in this ranking is phenylalanine. To investigate whether the high interfaciality of the tryptophans in the aromatic domain was important for the entry-mediating activity of the SARS S protein, we decided to replace one or more tryptophans for phenylalanine residues, creating mutants S_W1→F_, S_W12→F_, S_W123→F_, S_W2→F_, S_W23→F_, S_W3→F_, and S_W13→F_. Figure 2A shows that replacing all three tryptophan residues for phenylalanine (mutant S_W123→F_) resulted in a total lack of SARSpp entry. Also the mutant S proteins containing two out of three phenylalanine in stead of tryptophan (mutants S_W12→F_, S_W23→F_, and S_W13→F_) were not capable of mediating entry of SARSpp. Only the mutants containing one phenylalanine replacing a tryptophan (S_W1→F_, S_W2→F_, and S_W3→F_) had some residual entry-mediating activity (up to 30% for S_W2→F_). This clearly shows that the tryptophan residues in the aromatic domain of SARS S play a very specific role during entry of SARSpp that cannot be taken over by the phenylalanine residues. Therefore, the high propensity to participate in the interface between water and membrane is probably not the only feature of the tryptophan residues that is involved in mediation of SARSpp entry, although it cannot be ruled out at this time. To exclude the possibility of a defect in protein maturation or incorporation into SARSpp, all aromatic domain mutants were analyzed for trimerization and incorporation efficiency. All mutants were incorporated efficiently into SARSpp and were able to trimerize (Figures 2B and 2C).

### Aromatic-domain is involved in pore dilation

**Figure 3:**
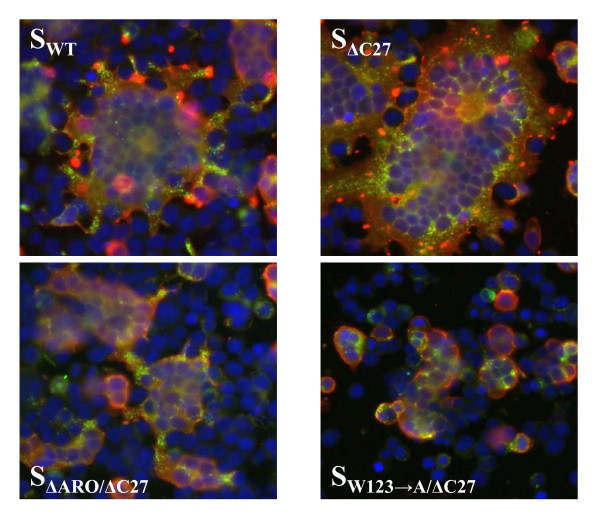
**Capacity of S proteins with mutations in the aromatic domain to mediate cell cell fusion or pore formation**. Cell cell fusion of 293T cells expressing ACE-2 and SARS CoV S, or S mutants. Cells were stained as described in the material and methods section. Red stain shows ACE-2 and green shows SARS CoV S protein. Cells expressing S_wt _or S_ΔC27 _clearly formed syncitia, whereas the mutants S_ΔARO_/ΔC27 and S_W123→A/ΔC27 _did not.

**Figure 4:**
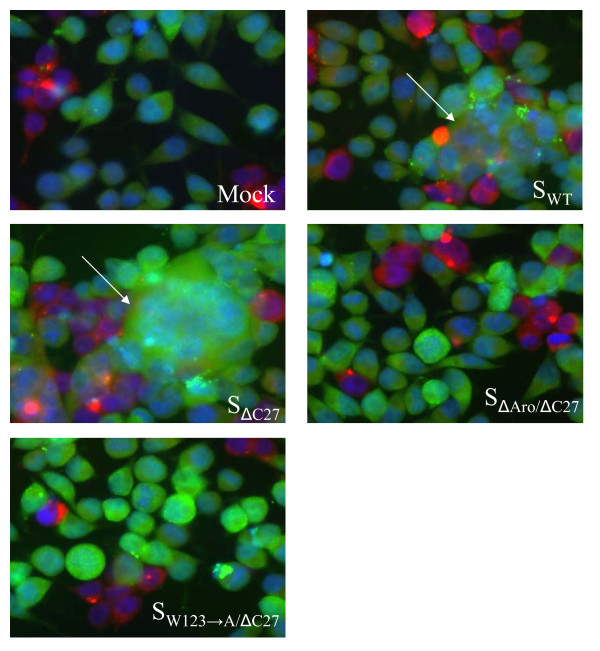
**Full cell cell fusion, as shown by exchange of intracellular dyes, unable to transfer spontaneously from one cell to another**. Double staining of a cell indicated full cell cell fusion. Pore formation does not result in dye exchange. L cells expressing ACE2 were stained green by CMFDA and 293T cells expressing (mutant) S were stained red by CMTPX.

Tryptophans play an important role during GP41 mediated fusion of the HIV membrane and its target membrane. In particular, tryptophan residues have been implicated in the process of fusion pore dilation, *i.e*. the enlargement of a fusion pore [7,8]. How this exactly works is poorly understood. We decided to test several mutants of the aromatic domain for their capacity to mediate cell-cell fusion and induce fusion pore formation. For these particular experiments, we decided to use mutants of the aromatic domain in the context of a deleted C-terminus (S_ΔC27_). This would yield a higher amount of S molecules on the surface of the cell, thereby potentially increasing the window of the cell-cell fusion assay [17]. Therefore, mutants S_ΔARO_/ΔC27 and S_W123→A/ΔC27 _were created and used in a cell-cell fusion experiment, together with SWT and S_ΔC27 _as controls. In Figure 3, pictures are shown of syncytia, representative for the cultures, induced by S or mutant S molecules. SWT clearly induced rounded syncytia consisting of approximately 50 cells, as determined by the number of nuclei. S_ΔC27 _was able to induce much larger, rounded syncytia, with at least twice as many nuclei as SWT, indicative for the high concentration of S_ΔC27 _at the surface of the cell. In contrast, syncytia induced by S_ΔARO_/ΔC27 and S_W123→A/ΔC27 _contained only a few cells and had an irregular shape, compared to SWT or S_ΔC27 _induced syncytia. Cells seemed to form small groups, but with a different morphology than the positive controls, indicating that the cell-fusion mediating activity was altered. Rather, the cells seemed to stick together, yet not to fuse. These results were confirmed by mixing L-ACE2 cells (L cells stably expressing human ACE2 after transfection with pFLACE2/T7RLuc [14,18] with 293T cells transiently expressing S or S mutants. To measure the mixing of the cell contents, L cells were stained green by CMFDA (Invitrogen) and 293T cells were stained red by CMTPX (Invitrogen). Since both these dyes are unable to transfer spontaneously from one cell to another, co-staining of cells is a measure for cell fusion. Large syncytia, staining positive for both dyes, were formed with SWT and S_ΔC27_, whereas no co-staining was observed with S_ΔARO_/ΔC27 and S_W123→A/ΔC27_, indicating a lack of fusion (Figure 4).

**Figure 5:**
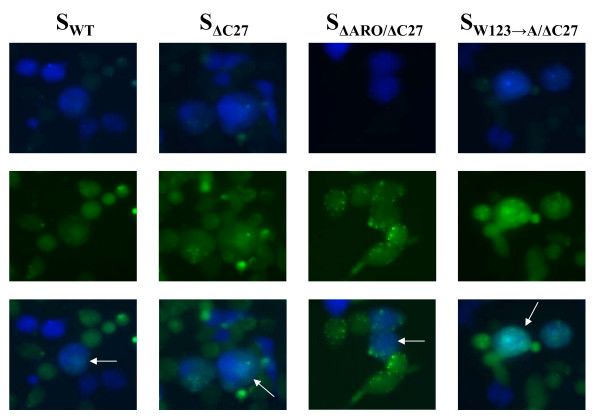
**Pore formation, as shown by the transfer of calcein**. Cells expressing ACE-2 (calcein loaded, green) and (mutant) S (CMAC loaded, blue) were mixed and transfer of calcein from the ACE-2 expressing cells to the S-expressing cells was monitored. Double staining indicated calcein transfer and thus the existence of fusion pores.

Previously, cells expressing aromatic-mutants of HIV-1 GP41 molecules have been shown to be unable to induce syncytium formation, but were shown to induce fusion pore formation, with a block in the dilation of the fusion pore, as evidenced by the transfer of small dye molecules from one cell to another [7,8]. We investigated the SARS S aromatic mutants for their capacity to display a similar activity, *i.e*. the induction of fusion pores, yet the incapability to establish full cell fusion. S-expressing cells were labeled with CMAC-blue, a dye that once it is present in the cytoplasm, will be modified by cytosolic enzymes, which makes it unable to transfer from one cell to another. ACE-2 expressing cells were labeled with calcein, which can be transferred from one cell to another through fusion pores. Double fluorescent cells thus represented the transfer of calcein from an ACE-2 expressing cell to an S-expressing cell, thereby showing the existence of fusion pores. In Figure 5, samples are shown of double fluorescent cells. As clearly shown, all mutants were able to form fusion pores since they all showed double fluorescent cells.

### Introduction of lysines in the SARS S TMD

**Figure 6:**
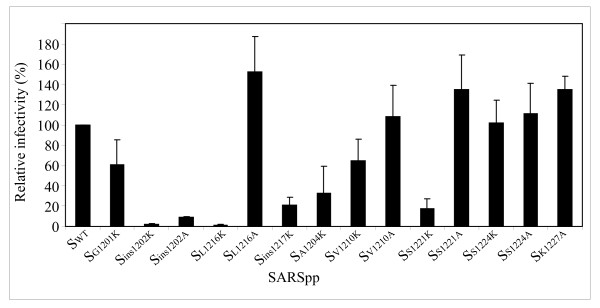
**Capacity of S proteins with lysine insertions or replacements in the TMD to mediate entry of SARSpp into VeroE6R**. Titers of the mutant S containing pseudoparticles are shown as a percentage of wild type S containing pseudoparticles.

As shown in Figure 1A, the supposed TMD domain of SARS S is quite long, especially when the locations of charged residues, that usually define the borders of a TMD, are considered. The N-terminal lysine (pos. 1193) is located in the aromatic domain, whereas the C-terminal lysine is located between cys box 1 and cys box 2 (pos. 1227). The number of amino acids between these two lysines is 33, whereas a typical TMD consists of approximately 16-20 amino acids. In an attempt to map the actual borders of the TMD of SARS S we constructed a range of S mutants in which extra lysine residues were introduced between the two previously mentioned lysines. (see Figure 1B) with the idea that introduction of a lysine in a hydrophobic (transmembrane) environment would result in a defective protein, unable to establish fusion. In addition, at two positions, we inserted lysines rather than replacing the existing amino acids for lysine. As controls, mutants with alanines in stead of the introduced lysines were made. When these S mutants where expressed, they could all be incorporated into SARSpp (results not shown) and all were able to form trimers (not shown), which indicates that all mutants matured correctly. However, the ability of some of the S mutants to mediate entry of the SARSpp was seriously decreased (see Figure 6).

Introducing a lysine directly downstream of the aromatic domain yielded two fundamentally different outcomes. When glycine 1201 was replaced for a lysine residue (SG1201K), S mediated entry was 60% compared to entry mediated by SWT, indicating that a charged residue could be tolerated at this position. However, inserting a lysine residue between G1201 and F1202 (Sins1202K) resulted in a complete block of entry. To check whether this phenotype was the result of the charge of the lysine, an alanine was inserted at the same position (Sins1202A). To our surprise, this mutant also had a very low capacity to mediate entry of SARSpp, indicating that not so much the charge of the lysine was detrimental for the activity of the spike protein, but rather the presence of an extra amino acid at that position.

Introducing a lysine directly upstream of cys box 1, either through replacement or insertion (mutants S_L1216K _and S_ins1217K_), resulted in a very low entry activity, indicating that at this position the charge of the lysine was not tolerated. Indeed, this was further confirmed by the SL1216A control mutant that was even more active than SWT. Introduction of lysines at the positions A1204, and S1221 resulted in a severe reduction in fusion mediating capacity (≤ 30%), whereas mutants SV1210K and S_S1224K _were hardly altered in their fusion mediating capacity. Both A1204 and S1221 are probably located in highly hydrophobic regions that do not tolerate a charged residue. Results with the S_V1210K _and S_S1224K _mutants suggest that the amino acids at these positions are in a less hydrophobic environment in which the charge is tolerated. This means that position 1210 is at the exact middle of the membrane, a place where charged residues can be tolerated [19]. Likewise, S1224, located downstream of the fourth cys residue of cys box 1, probably is not in the membrane, since the lysine was tolerated at that position. Altogether, we propose that the membrane spanning domain of SARS S starts at F1202 and ends at S1224.

## Discussion

The TMDs of viral fusion proteins are less well studied than their ectodomain counterparts. For a long time the TMDs have been appreciated solely for their anchoring function. However, it has become clear that the anchoring function of the TMDs is just one task. TMDs have now been implicated in virus assembly, protein sorting, oligomerization and fusion. Here, we report the importance of the TMD of the SARS CoV spike protein for mediating membrane fusion and entry.

### Lysine scanning mutagenesis

Mutagenesis by lysine insertion showed that at position 1201 a charged residue could be tolerated, indicating that the aromatic domain is located outside the membrane. Indeed, in HIV gp41, a lysine is present between the aromatic domain and the transmembrane domain. However, surprisingly, the lysine was tolerated only when it was a replacement and not an insertion. Further investigation showed that insertion of any amino acid at that position results in a non-functional S protein, which suggests that the positioning of the aromatic domain and the transmembrane domain relative to each other is crucial for membrane fusion activity of SARS S.

The fact that mutant SS1221K is incorporated in SARSpp implies that during maturation the cys box 1 does not need to enter a hydrophobic environment. However, it does not support fusion, suggesting that at some point after fusion activation, the cys box 1 is supposed to enter the viral membrane. This hints towards a model in which the long TMD has a dynamic nature which ensures that the lateral position of the helical TMD in the membrane can be varied, depending on the stage of the fusion process.

### Role of tryptophan residues during SARS CoV membrane fusion activity

The tryptophan mutants are severely crippled in their capacity to mediate SARSpp entry and induce cell-cell fusion, confirming data recently published [15,20]. The more tryptophans are lacking in the aromatic domain, the less active the spikes are in their entry-mediating capacity. In contrast, previously, mutant W1 → F was shown to be completely inactive (mutant W1194F, [20]), whereas we found approximately 17% activity. This difference cannot be explained, but it is conceivable that a single mutant would exhibit residual activity, as compared to double or triple mutants. In Figures 3, 4 and 5, we show that mutants that do not have tryptophans in the aromatic domain, are unable to support entry of SARSpp, yet are capable of initializing membrane fusion, *i.e*. capable of forming a fusion pore. The aromatic domain of HIV GP41 has been suggested to be involved in dilation of the fusion pore [7,8]. Saez-Cirion *et al*. have proposed two possible mechanisms by which the tryptophan residues might promote pore dilation. The first model is that the tryptophans enhance the transition between two lipidic stages of the fusion process. The second model is that the tryptophanes are involved in sequestering of multiple GP41 molecules to establish a proteinaceous ring thereby promoting the formation of a fusion pore [21]. Several studies are in support of the lipid model. Sainz Jr. *et al *have suggested that during the conformational changes in the spike the aromatic domain might align the fusion peptide and the TMD, thereby functioning as a hydrophobic sheet to allow lipid flow between the two fusing membranes [22]. Other reports have shown that peptides, representing the aromatic domain (of SARS CoV or HIV) are membrane active and are capable of altering the biophysical properties of membranes [21,23-25]. Furthermore, tryptophan residues have been shown to interact with cholesterol in the membrane, thereby modulating membrane curvature, possibly supporting lipidic intermediates during membrane fusion [26]. Both the HIV GP41 and SARS CoV S also contain a so-called CRAC motif (cholesterol recognition/interaction amino acid consensus) L/V-(X)_(1-5)_-Y-(X)_(1-5)_-R/K, located upstream of the transmembrane domain, which might also be of importance during membrane fusion activity [27-30]. The sequestering of the aromatic domain is supported by the study that showed oligomerization of HIV GP41 in solution [21]. Indeed, when expressed in bacteria, the SARS S aromatic domain forms hexamers in solution (J. Corver and W. Spaan, unpublished observation). It has also been shown that SARS CoV entry is dependent on the presence of lipid rafts [31], which are known to be enriched in cholesterol, arguing for the importance of cholesterol during SARS CoV entry.

In this paper, we show that the function of the aromatic domain of SARS CoV S is similar as the function of the HIV GP41 aromatic domain, as evidenced by the same phenotypic features of the mutants lacking tryptophan residues. Replacement of the tryptophans by phenylalanine residues resulted in a complete block of fusion (Figure 3, 4, 5), suggesting that not only the interfaciality of the tryptophans is important, but also other features. In addition, we found that the distance between the aromatic domain and the TMD is critical, since insertion of one amino acid resulted in a block of entry (Sins1202K and Sins1202A). This suggests that the positioning of the aromatic domain relative to the hydrophobic domain is crucial for membrane fusion activity, a feature that was not yet included in the model that tries to explain the role of the aromatic domain in membrane fusion activity of coronaviruses. We therefore like to propose a model into which this new characteristic has been added.

### Model

**Figure 7:**
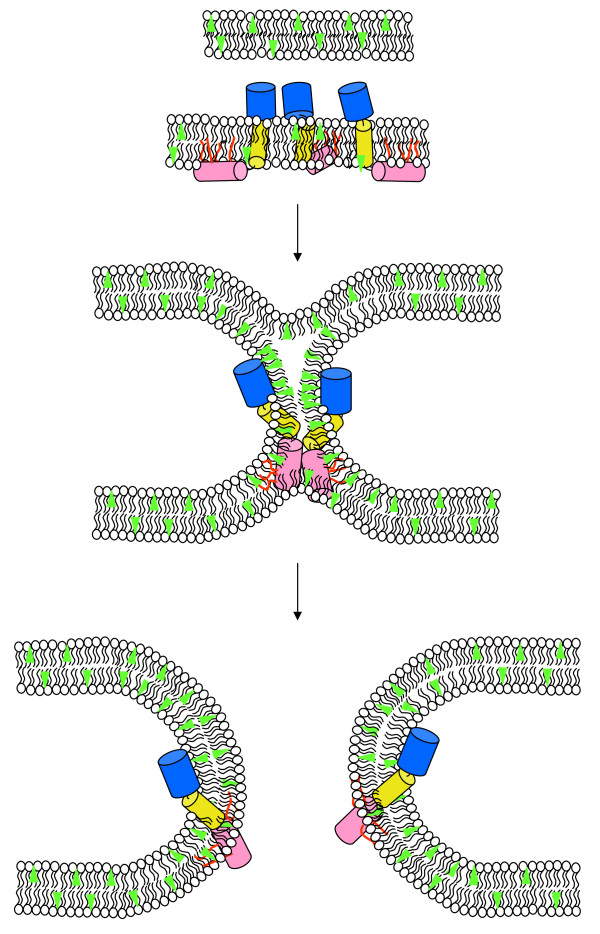
**Proposed model of membrane fusion, mediated by SARS S**. The model predicts the lateral flexibility of the TMD and the adjacent aromatic and cysteine rich domains to accommodate the necessary intermediates during membrane fusion. Blue, aromatic domain; yellow, hydrophobic core; pink, cysteine rich domain, green triangles, cholesterol.

Based on the results described above and on results published by others, we propose a mechanism in which the lateral flexibility of the aromatic domain, the TMD domain and the cys box 1 domain are pivotal for entry (membrane fusion activity) of SARSpp and most likely also SARS CoV. The model comprises the following steps (see Figure 7): i) In the native mature spike protein, the aromatic domain is folded into the ectodomain of the spike. Previously, it has been shown that the aromatic domain in native, prefusion GP41 is already associated to the interface of the viral membrane [32], forming the feet of the glycoprotein trimer tripod. However, the spikes on SARS CoV do not show these typical feet or a tripod like structure [33]. Therefore, it is possible that the position of the aromatic domain of SARS CoV S is different in the native conformation than in HIV GP41. The hydrophobic central TMD is in the membrane and the cys box 1 is outside the membrane, but closely associated to the membrane through the palmitic acids. ii) Once activated, the spike refolds and initiates fusion. It folds back to form the HR1-HR2 six helix bundle. As a result, the aromatic domain becomes associated to the membrane interface through its tryptophan residues, thereby pulling out the hydrophobic core of the TMD and thus iii) pulling in the cys box 1, which then partially becomes a transmembrane-like domain. Once released, the aromatic domain oligomerizes and consequently the S proteins cluster. The aromatic domain oligomerization also results in a clustering of cholesterol in the membrane, thereby inducing bending of the membrane with a negative curvature. Grouping of the S proteins, as induced by oligomerization of the aromatic domain, would also be necessary to establish pore dilation, *i.e*. the transition from a pore to a real fusion event, enabling large molecules to pass. Finally, as shown previously, the glycines present in the hydrophobic central domain of SARS CoV S also play an important role during entry [34], possibly by mediating bends in the TMD needed for membrane fusion pore formation [6]. Bending of the central hydrophobic helix would fit in the proposed model, enabling the positioning of the central domain during the lipidic intermediate stages (stalk and fusion pore).

## Conclusions

Based on the results in this paper we conclude that the aromatic rich region of the SARS CoV virus S protein plays a crucial role in the entry of SARS CoV. Most likely, as indicated in our model, the aromatic rich region is involved in pore dilation, possibly through interactions with cholesterol which modulate membrane curvature and could support lipidic intermediates during membrane fusion. In our model, we propose that the presence of the aromatic domain and the cysteine rich domain ensures a lateral flexibility of the S protein within the membrane, that is necessary for induction of membrane fusion. During this process, the positioning of the aromatic domain and the downstream amino acids relative to each other is another crucial prerequisite in the entry process of SARS CoV.

## Methods

### Cells and viruses

293T cells were obtained from the ATCC and cultured in DMEM with 10% FCS, VeroE6R cells were a kind gift of Dr. A.D.M.E. Osterhaus and they were cultured in DMEM with 10% FCS. SARS CoV strain Frankfurt was a kind gift of Drs H.F. Rabenau and H.W. Doerr.

### Plasmids

The plasmids encoding SARS spike (phCMV-SS) and ACE-2 (pFLACE-2/T7RLuc) were described before [14]. Plasmids encoding mutant spike proteins were created by using the Quikchange method (Stratagene) and the products were verified by sequencing.

### SARSpp synthesis and titer determination

Production of SARSpp was essentially carried out as described before [35]. Briefly, transfection of 293T cells with a set of retroviral Gag Pol expression constructs, a GFP reporter plasmid and the SARS CoV spike mutant to be expressed, was carried out using a CaCl_2 _transfection kit (Clontech). Two days after transfection supernatants were harvested and used to transduce VeroE6R cells. To correct for the amount of retroviral particles per transduction a C-type reverse transcriptase activity kit (Innovagen, Sweden) was used. Four days after transduction, the percentage of GFP positive cells was determined by FACS analysis on a FACS Calibur (Becton-Dickinson).

### Cell-cell fusion assays

Cell-cell fusion was measured in several assays. The basis for all assays was the same, only the read-out was different. For the immunofluorescence and dye transfer assay, 293T cells were transfected, using lipofectamine 2000 (Invitrogen) with plasmids encoding SARS S (or a mutant) or the SARS CoV receptor, ACE-2 [14]. Twenty four hours post transfection, cells were trypsinized, mixed and plated on cover slips. After 24 hours incubation, the assays were performed. The three different assays were: immunofluorescence assay (IFA), dye-transfer assay and cytoplasm mixing assay.

For the IFA, cells were mixed with a ratio of 1 to 4 (ACE-2 *vs *S respectively), fixed with 3.5% paraformaldehyde and permeabilized with 0.1% triton X-100. Subsequently, cells were incubated with goat anti ACE-2 antibody (R&D systems). Next, the cells were incubated with FITC conjugated swine-anti rabbit antibody (both from Sigma) and Hoechst. Then, the cells were incubated with rabbit-anti spike antibody (a kind gift from Dr. Eickmann) and Cy5 conjugated Rabbit-anti goat antibody. Finally, the cells were washed, mounted, and studied using a fluororescence microscope (Zeiss).

For the dye transfer assay, trypsinized, transfected cells were washed with PBS and resuspended in OptiMEM (Invitrogen). Subsequently, calcein (Molecular probes, Invitrogen) was added to the ACE-2 expressing cells to a final concentration of 2 μM and CMAC (Molecular probes, Invitrogen) was added to S expressing cells to a final concentration of 20 μM. Cells were incubated for 45 minutes at 37°C, while being resuspended every 15 minutes. Subsequently, media were refreshed and cells were transferred to clean tubes. After 30 minutes incubation at 37°C, cells were mixed in a 4 to 1 ratio (ACE-2 *vs *S expressing cells, respectively). Cells were then seeded on coated microscope slides (Clearcell, cel-line, Erie scientific company). After 24 hour incubation at 37°C, cells were fixed with 3.5% paraformaldehyde and analyzed using a fluorescence microscope (Zeiss).

For the cytoplasm mixing assay, L-ACE-2 cells, labeled with CMFDA (Invitrogen) as described for CMAC, were mixed with S-transfected 293T cells, labeled with CMTPX (Invitrogen) as described for CMAC. The ratio of the cells was 1 to 3 (S cells to ACE-2 cells, respectively). After 24 hours incubation, cells were fixed with 3.5% paraformaldehyde and analyzed by fluorescence microscopy.

## List of abbreviations

SARS: Severe Acute Respiratory Syndrome; CoV: Coronavirus; ACE: Angiotensin converting enzyme; FCS: Fetal Calf Serum; DMEM: Dulbecco's Modified Eagle Medium; FITC: Fluorescein isothiocyanate; IFA: Immuno Fluorescence Assay; FACS: Fluorescence-activated cell sorting; S: Spike; SARSpp: SARS pseudoparticles; TMD: Trans Membrane Domain; HIV: Human Immunodeficiency Virus.

## Competing interests

The authors declare that they have no competing interests.

## Authors' contributions

Design and conception of the study (JC, WS, RB); Experimental work (JC, RB, PvK); Writing of the manuscript (JC). All authors have read and approved the final manuscript.
